# Voluntary Exercise Prevents Hypertensive Response Sensitization Induced by Angiotensin II

**DOI:** 10.3389/fnins.2022.848079

**Published:** 2022-02-17

**Authors:** Baojian Xue, Jun-Ling Cui, Fang Guo, Terry G. Beltz, Zi-Gang Zhao, Geng-Shen Zhang, Alan Kim Johnson

**Affiliations:** ^1^Department of Psychological and Brain Sciences, The University of Iowa, Iowa City, IA, United States; ^2^Department of Neurosurgery, Second Hospital of Hebei Medical University, Shijiazhuang, China; ^3^Institute of Microcirculation, Hebei North University, Zhangjiakou, China; ^4^Department of Neuroscience and Pharmacology, The University of Iowa, Iowa City, IA, United States; ^5^Department of Health and Human Physiology, The University of Iowa, Iowa City, IA, United States; ^6^François M. Abboud Cardiovascular Research Center, The University of Iowa, Iowa City, IA, United States

**Keywords:** voluntary exercise, blood pressure, renin-angiotensin system, inflammation, central nervous system

## Abstract

Exercise training has profound effects on the renin-angiotensin system, inflammatory cytokines and oxidative stress, all of which affect autonomic nervous system activity and regulate blood pressure (BP) in both physiological and pathophysiological states. Using the Induction-Delay-Expression paradigm, our previous studies demonstrated that various challenges (stressors) during Induction resulted in hypertensive response sensitization (HTRS) during Expression. The present study tested whether voluntary exercise would protect against subpressor angiotensin (ANG) II-induced HTRS in rats. Adult male rats were given access to either “blocked” (sedentary rats) or functional running (exercise rats) wheels for 12 weeks, and the Induction-Delay-Expression paradigm was applied for the rats during the last 4 weeks. A subpressor dose of ANG II given during Induction produced an enhanced hypertensive response to a pressor dose of ANG II given during Expression in sedentary rats in comparison to sedentary animals that received saline (vehicle control) during Induction. Voluntary exercise did not attenuate the pressor dose of ANG II-induced hypertension but prevented the expression of HTRS seen in sedentary animals. Moreover, voluntary exercise reduced body weight gain and feed efficiency, abolished the augmented BP reduction after ganglionic blockade, reversed the increased mRNA expression of pro-hypertensive components, and upregulated mRNA expression of antihypertensive components in the lamina terminalis and hypothalamic paraventricular nucleus, two key brain nuclei involved in the control of sympathetic activity and BP regulation. These results indicate that exercise training plays a beneficial role in preventing HTRS and that this is associated with shifting the balance of the brain prohypertensive and antihypertensive pathways in favor of attenuated central activity driving sympathetic outflow and reduced BP.

## Introduction

Hypertension is major risk for cardiovascular disease affecting more than 31% adults worldwide. Uncontrolled hypertension is a risk factor for heart disease, renal failure, and stroke (Mills et al., 2020). The activation of the sympathetic nervous system (SNS) is a major mechanism both in human high blood pressure and in several models of hypertension in animals (DiBona, 2013; Dampney, 2016; Haspula and Clark, 2018; Johnson and Xue, 2018). In this regard, activation of renin-angiotensin system (RAS) and elevation of inflammation and oxidative stress in the central nervous system (CNS) produce a state of sympathetic overactivity that plays an essential role in the onset and development of hypertension (Peterson et al., 2006; Kalupahana and Moustaid-Moussa, 2012; Huber et al., 2017; Haspula and Clark, 2018). The brain regions involved in sympathetic activity and blood pressure (BP) regulation mainly are located in the basal forebrain including structures lying along the lamina terminalis [LT, the subfornical organ (SFO), the median preoptic nucleus (MnPO), and the vascular organ of the lamina terminalis (OVLT)] and the hypothalamic paraventricular nucleus (PVN; Johnson and Gross, 1993; Mimee et al., 2013; Sharma et al., 2021). Information reflecting the levels of blood-borne factors such as angiotensin (ANG) II, proinflammatory cytokines (PICs), reactive oxygen species (ROS), and extracellular sodium concentration sensed by the LT structures lacking a blood-brain barrier is projected through efferent pathways to the PVN. The PVN integrates this information and sends projections either to the rostral ventrolateral medulla (RVLM) or to the spinal cord intermediolateral cell column to exaggerate sympathetic outflow and elevate BP under the pathophysiological state of hypertension (Johnson and Gross, 1993; Mimee et al., 2013; Dampney, 2016; Sharma et al., 2021).

Exercise training of moderate intensity has been proven effective in preventing and managing cardiovascular diseases including heart failure and hypertension. The results of systematic reviews and meta-analysis of human participants indicate that endurance training, dynamic resistance training, and isometric resistance training significantly reduce diastolic BP. Endurance training might be most effective in reducing BP in hypertensive individuals (Cornelissen and Smart, 2013; Alpsoy, 2020). In the animal models of heart failure and hypertension, exercise training normalizes enhanced sympathetic activation and reduces elevated BP (Haack and Zucker, 2015; Michelini et al., 2015; Zucker and Musch, 2018). These beneficial effects of exercise training are associated with not only attenuation of the prohypertensive axis of the RAS [ACE1/ANG II/AT1-R; angiotensin-converting enzyme (ACE1), ANG II type 1 receptor (AT1-R)], PICs [tumor necrosis factor (TNF)-α, interleukin (IL)-1β, IL-6] and NADPH oxidase but also with improvement of the antihypertensive axis of the RAS [ACE2/ANG-(1-7)/Mas-R] and anti-inflammatory defense mechanisms (e.g., IL-10) in the brain regions including the RVLM and PVN (Agarwal et al., 2011; Zheng et al., 2012; Rossi et al., 2013; Masson et al., 2015; Zucker et al., 2015; Ferreira-Junior et al., 2019; Fragas et al., 2021).

Previous studies demonstrate that pretreatment with various challenges (stressors) resulted in hypertensive response sensitization (HTRS) to a subsequent pressor dose of ANG II (Johnson et al., 2015; Johnson and Xue, 2018; Xue et al., 2020b). The HTRS involves upregulated expression of RAS components, inflammatory markers (PICs and microglial activation) and NADPH oxidase in the LT and PVN induced by various challenges. Either central antagonism of the RAS or blockade of microglial activation or PIC production blocks the HTRS (Xue et al., 2012; Xue et al., 2016a,b; Xue et al., 2020b). Given that both the pathogenesis of cardiovascular disease and exercise training are associated with alterations in brain RAS activity, inflammation, and oxidative stress, we hypothesized that voluntary exercise training would protect against subpressor ANG II-induced HTRS *via* a similar central mechanism. To test this hypothesis, adult male rats were given access to either “blocked” (sedentary rats) or functional running (exercise rats) wheels for 12 weeks. The Induction-Delay-Expression (**I-D-E**) paradigm (Xue et al., 2012) was applied for the rats during the last 4 weeks of the sedentary vs. exercise conditions. During **I,** a low sub-pressor dose of ANG II or saline was delivered subcutaneously by osmotic minipump for 1 week. The rats then rested for 1 week (**D**), after which time, a second pump was implanted to deliver a slow-pressor dose of ANG II for 2 weeks (**E**). This experimental approach allowed assessment of the effect of voluntary exercise training on the induction of HTRS by pretreatment with a low sub-pressor dose of ANG II during **I** and expressed during **E** by administering a slow-pressor dose of ANG II. Running distances, metabolic markers and changes in BP were determined during voluntary running and **I-D-E** paradigm. Putative CNS molecular and cellular mediators including RAS components, inflammatory and oxidative stress markers in key brain structures (i.e., the LT and PVN) were also measured. The experimental results provide further evidence that exercise training blocks the sensitized hypertensive response through central mechanisms acting to alter activity in brain cardiovascular nuclei.

## Materials and Methods

### Animals

All experiments were conducted in accordance with the National Institutes of Health *Guide for the Care and Use of Laboratory Animals* and were approved by the University of Iowa and the Hebei Medical University Animal Care and Use Committee (Protocol # 1012115).

Forty Sprague-Dawley male rats (10-week-old, Envigo) were used in this study. All animals were maintained in a temperature (23 ± 2°C) and light (12-h light/dark cycle) controlled facility. The rats had unlimited access to standard rat chow and water.

### Voluntary Exercise and Sensitization Paradigm

**FIGURE 1:**
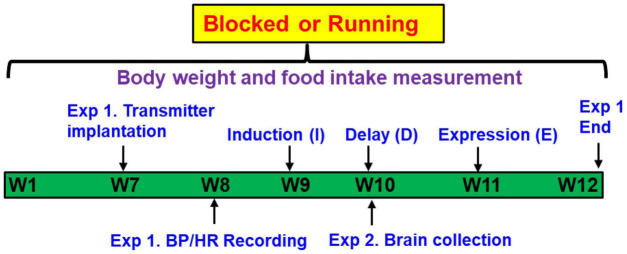
Representative timeline of the study design. Adult male rats were given access to either “blocked” or functional running wheels for 10–12 weeks. Experiment 1 (Exp. 1): the Induction-Delay-Expression (I-D-E) paradigm was applied for the rats during the last 4 weeks. The rats were pretreated during Induction with a systemic subpressor dose of angiotensin (ANG) II (sc, 10 ng/kg/min) for 1 week. After a 1-week Delay, rats were treated during Expression with a pressor dose of ANG II (120 ng/kg/min) for 2 weeks. The hemodynamic and autonomic function were measured. Experiment 2 (Exp. 2): at the end of Delay, animals were euthanized to collect brain tissues for assessing mRNA expression of renin-angiotensin system components, NADPH oxidase and proinflammatory cytokines.

Figure 1 shows the timeline of the study. Adult male rats were given access to either blocked (sedentary rats) or functional running (exercised rats) wheels equipped with activity monitors for 12 weeks, and the **I-D-E** paradigm was applied for the rats during the last 4 weeks, as described previously (Xue et al., 2012). During **I,** a low sub-pressor dose of ANG II or saline (S) was delivered subcutaneously (sc, 10 ng/kg/min) by osmotic minipump (model 2001, Alzet) for 1 week. The rats then rested for 1 week (**D**), after which time, a second pump (model 2002, Alzet) was implanted to deliver a slow-pressor dose of ANG II (120 ng/kg/min) for 2 weeks (**E**) to test for HTRS.

***Experiment 1.*** Rat transmitters (HD-S10, DSI^®^, St. Paul, MN, United States) were used to directly measure BP and heart rate (HR). At week 7, transmitters were implanted in both blocked and running animals. After 1-week recovery, baseline BP and HR recordings were made for 5 days, then the **I**-**D-E** paradigm was applied for all rats. Therefore, the experiments involved 4 groups (*n* = 5 rats/group): (1) Blocked **I**-S + **E**-ANG II; (2) Blocked **I**-ANG II + **E**-ANG II; (3) Running **I**-S + **E**-ANG II; and (4) Running **I**-ANG II + **E**-ANG II.

***Experiment 2.*** Additional studies were performed to assess the effects of the sedentary or exercise conditions on mRNA expression of RAS and PIC components and NADPH oxidase in the LT and PVN. Brains (*n* = 5 rats/group) were collected at end of **D**, corresponding to the time at which the pressor dose of ANG II infusion was initiated in Experiment 1.

### Running Distance and Metabolic Parameter Measurement

Voluntary running distance was recorded daily, and food intake and body weight were measured once a week in both sedentary and exercise rats. Running distance, food intake and body weight gain were averaged daily. Daily calorie intake was calculated: average daily food intake x diet energy density (standard rat chow, 3.85 kcal/g). Feed efficiency, the capacity to convert caloric intake into body weight, was determined by mean body weight gain (mg)/total caloric intake (kcal).

### Telemetry Transmitter and Osmotic Pump Implantation

The rats were anesthetized with a ketamine-xylazine mixture (90% ketamine and 10% xylazine), and the femoral artery was accessed with a ventral incision. The right femoral artery was isolated, and the catheter of a telemetry probe was inserted into the vessel. Through the same ventral incision, a pocket along the right flank was formed. The body of the telemetry transmitter (HD-S10, DSI^®^, St. Paul, MN, United States) was slipped into the pocket and secured with tissue adhesive. The ventral incision was then closed with suture. Beginning 7 days after surgery, BP and HR data collection was initiated.

In separate procedures under isoflurance anesthesia (0.5–5% inhalation), osmotic pumps (model 2001 or 2002, ALZET^®^, Cupertino, CA, United States) containing ANG II (10 ng/kg/min or 120 ng/kg/min, Sigma) were implanted subcutaneously in the back of rats, respectively.

### Evaluation of Blood Pressure Responses to Autonomic Blockade

Blood pressure was also measured in the presence of the ganglionic blocker hexamethonium (Hex, 30 mg/kg, ip). Ganglionic blockade was repeated two times in each animal once during baseline and once after 14 days of ANG II infusion. On the day of ganglionic blockade experiments, rats were allowed to stabilize for at least 60 min, after which time BP was recorded for 20 min before and after Hex injection.

### Real-Time PCR Analysis

In the experiment 2, all rats were decapitated, and the brains were quickly removed and put in iced saline for 1 min. Then, the brains were cut into coronal sections of approximately 200 μm thickness, and LT and PVN tissues were punched with a 15-gauge needle stub (inner diameter: 1.5 mm). Some immediately surrounding tissue was usually included in the punch biopsies. The structures lying along the LT include the SFO, MnPO, and OVLT. Because each of the structures lying along the LT is very small, we collected these structures together and analyzed their mRNA expression as a whole. The PVN is composed of both magnocellular parvocellular components. We collected magnocellular and parvocellular regions from both sides. Total RNA was isolated from the LT or PVN using the Trizol method (Invitrogen) and treated with DNase I (Invitrogen, Carlsbad, CA, United States) to remove any genomic DNA contamination. RNA integrity was checked by gel electrophoresis. Total RNA was reverse transcribed following the manufacturer’s instructions (Applied Biosystems, Foster City, CA, United States). Real time PCR was conducted using 200–300 ng of cDNA and 500 nM of each primer in a 20 μl reaction with iQ SYBR Green Supermix (Bio-Rad, Hercules, CA, United States). Amplification cycles were conducted at 95°C for 3 min, followed by 40 cycles of 95°C for 15 s and annealing/extension at 60°C for 30 s. Reactions were performed in duplicate and analyzed using a C1000 thermocycler system (Bio-Rad). Messenger RNA levels for RAS components (ACE1, AT1-R, AT2-R, Ang-(1-7) receptor Mas-R), PICs (TNF-α, IL-1β, IL-6 and IL-10), NADPH oxidase (NOX2) and GAPDH were analyzed with SYBR Green real-time RT-PCR. The values were corrected by GAPDH, and the final concentration of mRNA was calculated using the formula *x* = 2^–ΔΔCt^, where *x* = fold difference relative to control. Primers were purchased from Integrated DNA Technologies (Coralville, IA, United States). The sequences of the primers are shown in Table 1.

**TABLE 1:** Primer sequences for real time PCR.

Gene	Forward primer	Reverse primer	Product size (bp)
GAPDH	TGACTCTACCCACGGCAAGTTCAA	ACGACATACTCAGCACCAGCATCA	141
ACE	GTGTTGTGGAACGAATACGC	CCTTCTTTATGATCCGCTTGA	187
AT1-R	CTCAAGCCTGTCTACGAAAATGAG	GTGAATGGTCCTTTGGTCGT	188
NOX2	CAAGATGGAGGTGGGACAGT	GCTTATCACAGCCACAAGCA	170
TNF-α	GCCGATTTGCCACTTCATAC	AAGTAGACCTGCCCGGACTC	209
IL-6	GCCTATTGAAAATCTGCTCTGG	GGAAGTTGGGGTAGGAAGGA	160
IL-1β	AGCAACGACAAAATCCCT GT	GAAGACAAACCGCTTTTCCA	209
IL-10	GTTGCCAAGCCTTGTCAGAAA	TTTCTGGGCCATGGTTCTCT	120
AT2-R	ACCTTTTGAACATGGTGCTTTG	TTTCCTATGCCAGTGTGCAG	160
Mas-R	TGTGGGTGGCTTTCGATT	CCCGTCACATATGGAAGCAT	159

*ACE, angiotensin converting enzyme 1; AT1-R, angiotensin II type 1 receptor; NOX2, NADPH oxidase 2; TNF-α, tumor necrosis factor-α; IL-6, interleukin-6; IL-1β, interleukin-1β; IL-10, interleukin-10; AT2-R, angiotensin II type 2 receptor; and Mas-R, angiotensin-(1–7) receptor.*

### Statistical Analysis

Mean arterial pressure (MAP), HR and locomotor activity (relative animal activity movement) obtained from the telemetry recordings are presented as mean daily values averaged from daytime and nighttime measurements. Differences for MAP and HR were calculated for each animal based on the mean of a 5-day baseline subtracted from the mean of the final 5 days of ANG II treatment. For experiments of the effect of acute Hex injection, differences for BP were calculated for each animal based on the baseline subtracted from the BP after ip injection of Hex. All data were checked for the normality assumption by using Shapiro–Wilk test. One-way or Two-way ANOVAs for the experimental groups were then conducted on the means of the calculated differences for MAP and HR. After establishing a significant ANOVA, *post-hoc* analyses were performed with Tukey multiple comparison tests between pairs of mean changes (Graph-pad Prism 9.0). Running distance, body weight gain, food intake and feed efficiency are also presented as mean daily values and averaged daily values of the 12 weeks recordings. One-way ANOVAs were used to test for the differences in body weight gain, food intake, feed efficiency, and mRNA expression of the RAS and PIC components and NADPH oxidase in the LT and PVN, respectively. Running distances was analyzed by *t*-test. All data are expressed as means ± SEM. Statistical significance was set at *p* < 0.05.

## Results

### Voluntary Exercise Intensity in Rats

**FIGURE 2:**
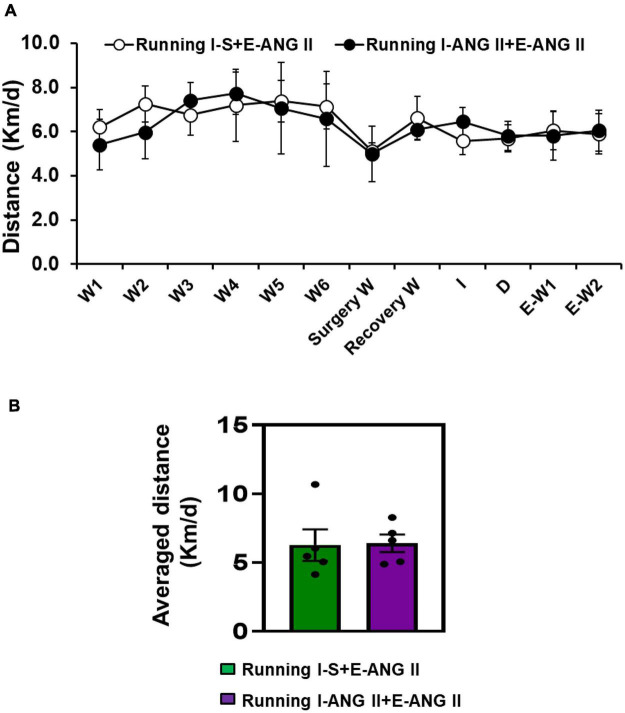
The daily **(A)** and averaged **(B)** running distances during 12 weeks of voluntary exercise (Running) in rats with Induction-Delay-Expression (I-D-E) paradigm.

The transmitter implantation surgery and I-D-E paradigm had no effects on exercise intensity in rats. The 12 weeks of running distances were comparable between two running groups [*t*-test, *P* = 0.9628] (Figures 2A,B).

### Effect of Voluntary Exercise on Metabolic Parameters in Rats

**FIGURE 3:**
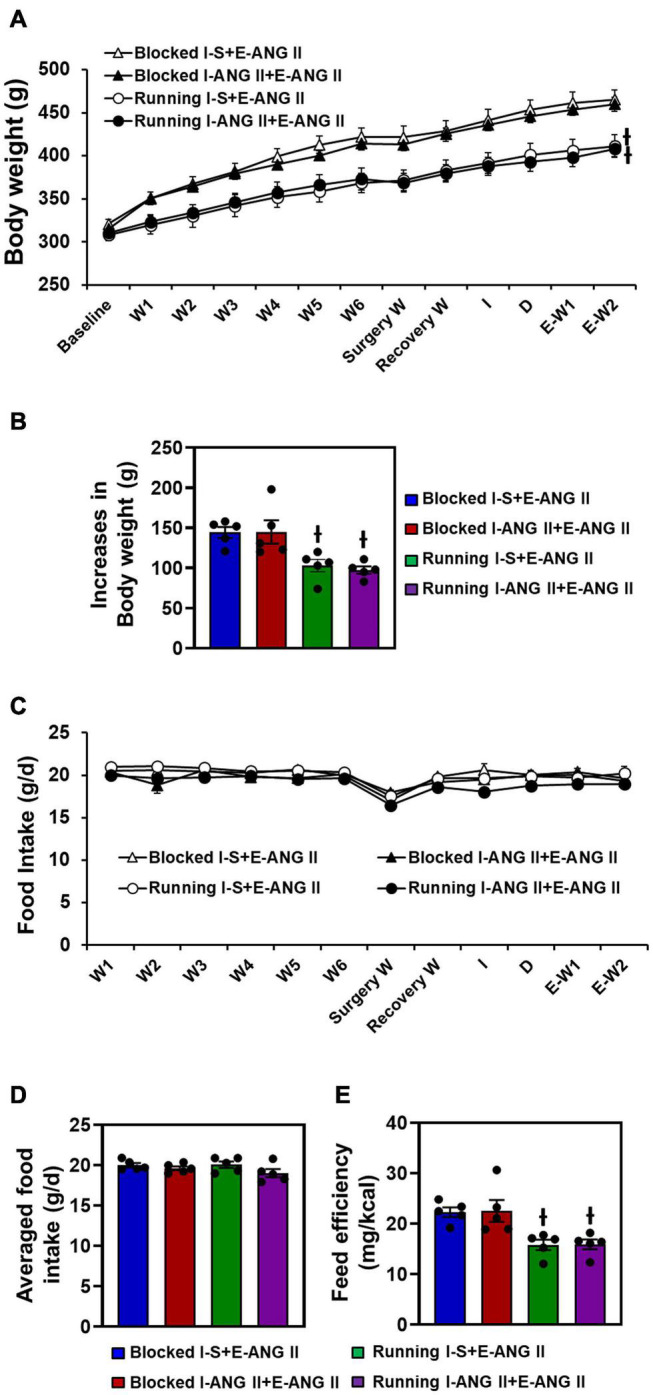
Body weight changes **(A,B)**, daily and averaged food intakes **(C,D)** and feeding efficiency **(E)** in sedentary (Blocked) and exercise (Running) rats with Induction-Delay-Expression (I-D-E) paradigm. (*n* = 5/group; one-way ANOVA was used for analysis followed by Tukey’s *post-hoc* tests. *p* < 0.05; -I vs. blocked rats).

Pretreatment with saline or the subpressor dose of ANG II and subsequent pressor dose of ANG II had no effects on body weight changes. After 12 weeks of voluntary exercise, the animals with running wheel access had significantly reduced body weight gain when compared to that of sedentary rats (One-way ANOVA analysis: *F*_(3,16)_ = 7.909, *P* = 0.0019) (Figures 3A,B).

There were no differences in food intake (g/d) between sedentary and exercise rats (*F*_(3,16)_ = 1.710, *P* = 0.2501). Due to the reduced body weight gain in exercise animals, feed efficiency was significantly lower in the exercise rats when compared to the sedentary rats (One-way ANOVA analysis: *F*_(3,16)_ = 7.569, *P* = 0.0023) (Figures 3C–E).

### The Effects of Voluntary Exercise on Locomotor Activity and Sensitization of Hypertension

**FIGURE 4:**
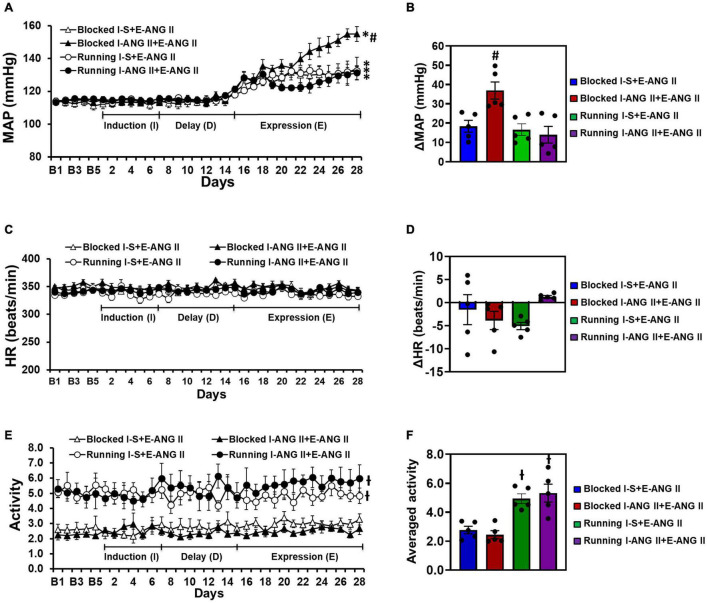
Voluntary exercise prevented hypertensive response sensitization to angiotensin (ANG) II treatment although the locomotor activities of the rats were elevated. HRs were comparable in all groups of offspring. Daily and averaged mean arterial pressure (MAP, **A,B**), heart rate (HR, **C,D**) and locomotor activity **(E,F)** in sedentary (Blocked) or exercise (Running) rats during the Induction-Delay-Expression (I-D-E) paradigm. (*n* = 5/group; two-way ANOVA was used for analysis followed by Tukey’s *post-hoc* tests. *p* < 0.05, * vs. baseline; # vs. blocked I-S + E-ANG II and running rats; -I vs. blocked rats).

In sedentary rats, pretreatment with a subpressor dose of ANG II during **I** resulted in an enhanced increase in MAP induced by subsequent infusion of pressor dose of ANG II when compared with the rats pretreated with saline during **I** (Δ:36.9 ± 4.4 vs. Δ:18.4 ± 3.0 mmHg, Two-way ANOVA analysis: *F*_(1,32)_ = 82.21, *P* < 0.0001). Voluntary exercise did not alter the slow-pressor dose of ANG II-induced hypertension in rats pretreated with saline during **I** (Δ:16.6 ± 3.0 mmHg). However, exercise abolished the effect of the subpressor dose of ANG II given to induce HTRS (Δ:14.0 ± 4.4 mmHg) (Two-way ANOVA analysis: *F*_(3,32)_ = 4.859, *P* = 0.0068, Figures 4A,B).

Exercise or infusion of ANG II did not produce significant changes in HR in any of the groups (Two-way ANOVA analysis: *F*_(3,12)_ = 1.681, *P* = 0.2238) (Figures 4C,D).

The I-D-E paradigm itself had no effect on locomotor activity in either the sedentary or exercise rats. However, the exercise rats exhibited a significant increase in locomotor activity (4.94 ± 0.34 and 5.32 ± 0.62) when compared with the sedentary rats (2.77 ± 0.26 and 2.45 ± 0.27) (One-way ANOVA analysis: *F*_(3,16)_ = 13.61, *P* = 0.0001, Figures 4E,F).

### Effects of Autonomic Blockade on Blood Pressure

**FIGURE 5:**
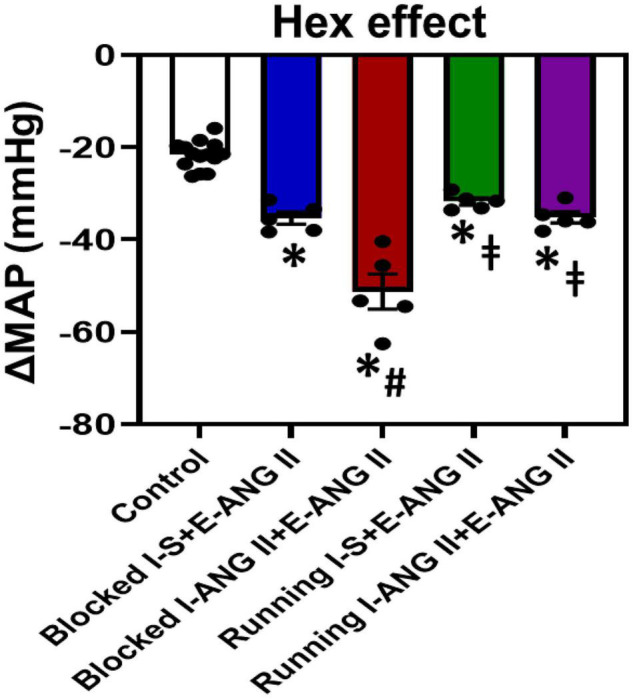
Mean arterial pressure (MAP) in response to ganglionic blockade with hexamethonium (Hex) before starting the Induction-Delay-Expression (I-D-E) paradigm (control) and at the end of the pressor angiotensin (ANG) II infusion in all groups. (*n* = 5/group; one-way ANOVA was used for analysis followed by Tukey’s *post-hoc* tests. *p* < 0.05, * vs. control, # vs. Blocked I-S + E-ANG II, vs. Blocked I + ANG II + E-ANG II).

Figure 5 shows the decreases in BP with acute ganglionic blockade in all groups. The average reduction in the BP response to Hex injection before the **I-D-E** paradigm was −21.7 ± 0.7 mmHg. Following 14 days of the slow-pressor dose of ANG II, acute Hex injection resulted in a significant reduction in BP in sedentary rats pretreated with saline during **I** (Δ:−35.4 ± 1.3 mmHg, *P* = 0.0023). However, the reductions in BP after Hex injection were further augmented in sedentary rats pretreated with the subpressor dose of ANG II during **I** (Δ:−51.3 ± 3.8 mmHg, *P* = 0.0006). Voluntary exercise did not alter Hex-induced reduction in rats pretreated with saline during **I** when compared to the corresponding sedentary rats (Δ:−31.7 ± 0.8 mmHg, *P* = 0.7529). However, exercise abolished the augmented reduction in BP induced by Hex in rats pretreated with subpressor dose of ANG II during **I** (Δ:−35.2 ± 1.2 mmHg, *P* = 0.0006) (One-way ANOVA analysis: *F*_(4,29)_ = 51.93, *P* < 0.0001, Figure 5).

### The Effects of Subpressor Dose of Angiotensin II and Voluntary Exercise on mRNA Expression of Brain Renin-Angiotensin System Components, Proinflammatory Cytokines, and NADPH Oxidase

**FIGURE 6:**
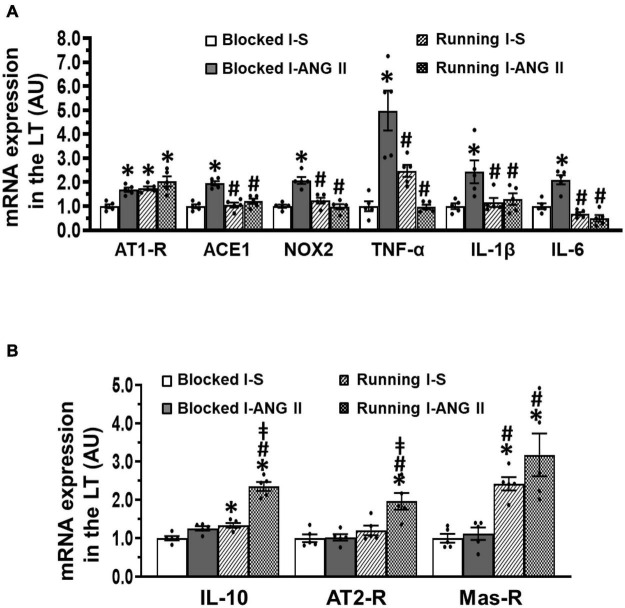
Comparison of the mRNA expression of renin-angiotensin system components, NADPH oxidase and proinflammatory cytokines in the lamina terminalis (LT) in sedentary (Blocked) or exercise (Running) rats after Delay period of the Induction-Delay-Expression (I-D-E) paradigm. **(A)** mRNA expression of prohypertensive components; **(B)** mRNA expression of antihypertensive components (*n* = 5/group; one-way ANOVA was used for analysis followed by Tukey’s *post-hoc* tests; *p* < 0.05; * vs. Blocked I-S; # vs Blocked I-ANG II; vs. Running I-S).

In LT tissues of the sedentary rats collected at the end of **D**, RT-PCR analysis revealed that subpressor dose of ANG II pretreatment during **I** resulted in a significant increase in mRNA expression of all prohypertensive components including AT1-R, ACE, NOX2, TNF-α, IL-1β, and IL-6 (*P* < 0.05, Figure 6A), but did not alter mRNA expression of antihypertensive components such as IL-10, AT2-R, and Mas-R when compared with saline pretreatment (*P* > 0.05, Figure 6B). Voluntary exercise reversed the increased mRNA expression of most of prohypertensive components except AT1-R and produced significant increase in mRNA expression of antihypertensive components (*P* < 0.05, Figures 6A,B). Notably, voluntary exercise in subpressor ANG II pretreated rats exhibited an augmented increase in mRNA expression of IL-10 and AT2-R when compared to the exercise rats pretreated with saline during **I** (*P* < 0.05, Figure 6B). (One-way ANOVA for the changes in gene expression, *P-*values from 0.0155 to less 0.0001).

**FIGURE 7:**
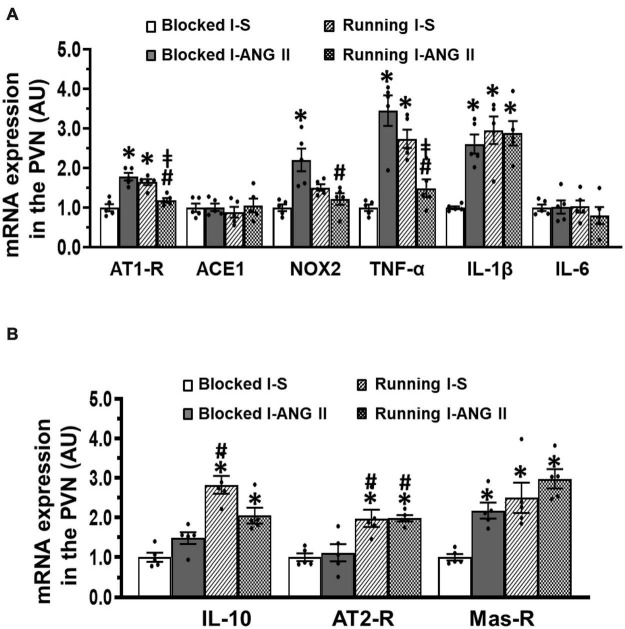
Comparison of the mRNA expression of renin-angiotensin system components, NADPH oxidase and proinflammatory cytokines in the hypothalamic paraventricular nucleus (PVN) in sedentary (Blocked) or exercise (Running) rats after Delay period of the Induction-Delay-Expression (I-D-E) paradigm. **(A)** mRNA expression of prohypertensive components; **(B)** mRNA expression of antihypertensive components (*n* = 5/group; one-way ANOVA was used for analysis followed by Tukey’s *post-hoc* tests; *p* < 0.05; * vs. Blocked I-S; # vs. Blocked I-ANG II; vs. Running I-S).

In PVN tissues, the sedentary rats pretreated with the subpressor dose of ANG II during **I** exhibited upregulation of mRNA expression of the prohypertensive components (AT1-R, NOX2, TNF-α, IL-1β, *P* < 0.05) and an antihypertensive component Mas-R (*P* < 0.05), but had no effects on mRNA expression of the ACE1, IL-6, IL-10, and AT2-R (*P* > 0.05, Figures 7A,B). The increased expression of AT1-R and TNF-α was not altered in saline pretreated rats after voluntary exercise (*P* > 0.05). However, the increased expression of AT1-R, NOX2 and TNF-α was not present in the subpressor ANG II pretreated exercise rats (*P* < 0.05). Moreover, voluntary exercise in both saline and subpressor ANG II pretreated rats upregulated mRNA expression of antihypertensive components IL-10 and AT2-R (*P* < 0.05) and did not change the increased expression of IL-1β and Mas-R induced by subpressor ANG II pretreatment in sedentary rats (*P* > 0.05) (Figures 7A,B). (One-way ANOVA for the changes in gene expression; ACE1, *P* = 0.8126; IL-6, *P* = 0.7333; other *P*-values from less 0.0008 to less 0.0001).

## Discussion

The goals of the current studies were to determine if voluntary exercise would attenuate the sensitized hypertensive response induced by ANG II infusion and to investigate if it was through regulating the balance between the prohypertensive and the antihypertensive pathways in the CNS. The major findings of the present study were that (1) a systemic subpressor dose of ANG II pretreatment elicited HTRS to a subsequent infusion of a slow-pressor dose of ANG II in sedentary rats, which were mediated by increased centrally driven sympathetic tone. However, this augmentation of the hypertensive response and sympathetic tone was not exhibited by the exercised rats; (2) although there were similar food intakes between the sedentary and exercise rats, the exercise rats showed reduced body weight gain and feed efficiency; and (3) the subpressor dose of ANG II infusion during **I** resulted in significant increases in mRNA expression of several prohypertensive components such as AT1-R, ACE1, NOX2, TNF-α, IL-1β, and IL-6 in key brain cardiovascular nuclei when measured at the end of **D**. Exercise abolished the upregulated expression of most of the prohypertensive components, and upregulated mRNA expression of some antihypertensive components including IL-10, AT2-R, and Mas-R. The results indicate that exercise training plays a beneficial role in preventing HTRS and that this is associated with reduced expression of prohypertensive components of the RAS, PICs, and NADPH oxidase and with enhanced expression of antihypertensive components of the RAS and anti-inflammatory cytokine in the CNS. This study highlights a central mechanism by which exercise training mitigates an enhanced increase in BP and the development of hypertension when one encounters prohypertensive stressors in the lifetime.

The sympathetic nervous system (SNS) plays a pivotal role in the development and maintenance of hypertension in human and several animal models, including ANG II-induced hypertension, obesity-related hypertension, deoxycorticosterone acetate/aldosterone salt-sensitive hypertension, and spontaneously hypertensive rats (SHRs; Shi et al., 2010a; Xue et al., 2011; Cardinale et al., 2012; Chao et al., 2013; Dange et al., 2015; Hall et al., 2015; Basting and Lazartigues, 2017). In this regard, increased brain ANG II, PICs and ROS derived either from *de novo* synthesis in the brain or from the circulation and upregulated expression of their receptors or genes (i.e., AT1-R, ACE, and TNF receptors) are evident in these hypertensive animals (Young and Davisson, 2015; Nakagawa et al., 2020). Interactions between RAS, PICs and ROS activate the brain neural network containing neurons in the LT and PVN to elevate the SNS activity and increase BP (Shi et al., 2010a; Xue et al., 2011; Cardinale et al., 2012; Chao et al., 2013; Dange et al., 2015; Hall et al., 2015; Young and Davisson, 2015; Basting and Lazartigues, 2017; Nakagawa et al., 2020). Previous studies from our laboratory have also demonstrated that a wide range of stressors sensitize the hypertensive response through upregulation of these prohypertensive factors in the brain (Johnson and Xue, 2018; Xue et al., 2020b). In the present study, the subpressor dose of ANG II infusion during **I** resulted in significant increases in mRNA expression of prohypertensive components such as AT1-R, ACE1, NOX2, TNF-α, IL-1β, and IL-6 in the LT and PVN of the sedentary rats. These upregulated prohypertensive components enhancing CNS activity may provide a physiological bases for initiating and maintaining a sensitized state. As a result, the enhanced hypertensive response to the subsequent pressor dose of ANG II and augmented sympathetic tone were evident in the sedentary rats pretreated with subpressor dose of ANG II.

Exercise training is an effective strategy for maintaining health or rehabilitating subjects who are deconditioned due to cardiovascular disease (Zucker and Musch, 2018; Alpsoy, 2020). The mechanisms underlying the beneficial effects of exercise training have been investigated extensively in human and animal models of various cardiovascular diseases such as hypertension, heart failure and stroke. Central mechanisms including inhibition of RAS activity, reduction of microglial activation, inflammation and oxidative stress, and restoration of the blood-brain barrier in brain cardiovascular nuclei such as the PVN and RVLM have been demonstrated to result from exercise training. Exercise effectively reduces SNS activity and BP and improves heart failure (Agarwal et al., 2011; Zheng et al., 2012; Haack and Zucker, 2015; Masson et al., 2015; Michelini et al., 2015; Ferreira-Junior et al., 2019; Fragas et al., 2021). In the present study, we found that voluntary exercise normalized the subpressor dose of ANG II-induced upregulation of mRNA expression of several RAS components and PICs and NADPH oxidase in the LT and PVN and that these were accompanied by the abolition of HTRS and attenuation of increased sympathetic tone. Our present results confirm and extend the point that exercise training blocks the development of hypertension produced by the sensitized hypertensive response through inhibiting central prohypertensive factors and reducing sympathetic outflow.

Besides the prohypertensive components of the renin-angiotensin and immune systems, there are anti-hypertensive and anti-inflammatory components including ACE1/ANG II/AT2-R, ACE2/ANG-(1-7)/Mas-R, and IL-10 both in the CNS and in the periphery. The anti-hypertensive components oppose the actions of the prohypertensive axis of RAS (Shi et al., 2010a; Gao and Zucker, 2011; Xu et al., 2011; Sumners et al., 2015; Santos et al., 2018). Reduced AT2-R or ACE2 expression and/or enzyme activity have been found in various brain regions in hypertension and heart failure models. Overexpression or activation of AT2-R, ACE2, or Mas-R in the brain blunts the development of hypertension and improves heart failure (Gao et al., 2008; Feng et al., 2010; Xia et al., 2013). As an anti-inflammatory factor, IL-10 has a significant impact on sympathetic outflow, BP and cardiac remodeling in experimental models of hypertension (Shi et al., 2010b). Studies show that IL-10 can inhibit microglial activity and prevent lipopolysaccharide (LPS)-induced production and secretion of PICs through the toll-like receptor-4 (TLR4) and the nuclear factor kappa B (NF-κB) signaling cascade (Mee-Inta et al., 2019).

It has been demonstrated that long-term exercise training can upregulate IL-10, ACE2, and Mas-R expression in the brain of SHR and heart failure models (Agarwal et al., 2011; Zucker et al., 2015). Consistent with these previous studies, we also found in the present study that exercise training upregulated the mRNA expression of antihypertensive components including IL-10, AT2-R, and Mas-R in the LT and PVN of sedentary animals and induced an enhanced mRNA expression of these antihypertensive components in the LT in rats pretreated with the subpressor dose of ANG II. All these effects would tend to contribute to the attenuation of the subpressor dose of ANG II-induced increase in sympathetic tone and sensitization of hypertension. Our present results indicate that exercise training plays a protective role in the induction of HTRS not only through inhibition of prohypertensive components but also through activation of antihypertensive components of the RAS and immune system that rebalance activity of central neural network involved in regulation of BP.

Besides the effects on the autonomic nervous system, exercise training also impacts energy metabolism. In fact, the reduction of body weight and visceral adiposity, and subsequently, influence of metabolic related hormones such as leptin and insulin by exercise may interfere with the central mechanisms contributing to the development of hypertension, since high blood pressure has been associated with all these variables (Jakicic et al., 2001; Lim et al., 2013). In the present study, we found that the exercise rats showed reduced body weight gain and feed efficiency even though food intakes were similar between the sedentary and the exercise rats. Moreover, the reduced body weight and the distances run by the animals pretreated with either saline or a subpressor dose of ANG II were comparable. However, exercise did not affect the hypertensive response to a pressor dose of ANG II in saline pretreated animals, but abolished the sensitized hypertensive response induced by pretreatment with a suppressor dose of ANG II. This excludes the possibility that exercise-induced abolition of HTRS observed in this study was due to a reduction in body weight. However, the roles of exercise-induced changes in body composition, metabolic hormones (i.e., leptin and insulin) as well as their interactions with the RAS components and mediators of inflammation and oxidative stress in the sensitization of hypertension warrant further investigation. In addition, voluntary exercise has been demonstrated to induce less stress when compared with those forced exercise such as forced treadmill running or swimming (Billman et al., 2015). Forced exercise was not chosen as the method for this study because the psychological stress has been demonstrated to induce the HTRS in our previous studies (Xue et al., 2019; Xue et al., 2020a).

There are some limitations to this study. First, besides changes in the neural network that regulates SNS and BP, the beneficial effects of exercise training in cardiovascular disease are also due to a large interplay of cellular and molecular mediators in the heart and peripheral vasculature (Zucker and Musch, 2018). It has been shown that long-term exercise plays a beneficial role in attenuating the transition from the pre- to hypertensive phase in SHRs through deactivation of the AT1-R pathway driving vascular and heart remodeling (Silva et al., 2015; da Costa et al., 2020). Based on these data, in our hypertension sensitization model, we can’t rule out the possibility that exercise training also affected vascular and heart function directly to attenuate the HTRS induced by pretreatment with subpressor dose of ANG II. Second, we found that pretreatment with a subpressor dose of ANG II upregulated mRNA expression of AT1-R in both the LT and PVN. However, voluntary exercise reversed upregulated mRNA expression of AT1-R only in the PVN, but not in the LT. The results may reflect the differences in the brain location between these two structures (outside vs. inside blood–brain barrier) or in the changes in mRNA expression of related RAS component such as ACE1 (changed in the LT but not in the PVN). This difference in the mRNA expression needs to be explored by measurements of enzymatic activity or protein expression. Therefore, more studies assessing the enzymatic activity, protein levels and activation of second messenger mechanisms related to these agents in these brain nuclei are needed to confirm the functional significance of the changes in gene expression. Third, it has been shown that interplays between sex hormones and exercise have a long-term regulatory effect on BP through affecting food intake and energy balance (Gentry and Wade, 1976; Yang and Liang, 2018). In our previous study, we also found sex differences in HTRS, in which pretreatment with subpressor dose of ANG II did not elicit HTRS in intact female rats when compared to male rats and ovariectomized female rats, and females are protected from ANG II-induced sensitization through central estrogen and its regulation of brain RAS (Xue et al., 2014). Based on these results, we did not include females in the present study to explore the beneficial effect of exercise on HTRS.

## Conclusion

This study demonstrated that in sedentary animals, pretreatment with a subpressor dose of ANG II induces HTRS probably through shifting the balance of the prohypertensive and antihypertensive pathways in favor of enhanced central activity driving sympathetic outflow and elevated BP. The voluntary exercise-induced blockade of the prohypertensive components or activation of the antihypertensive components leads to attenuated overall activity of the RAS and reduced inflammation and oxidative stress that is effective in lowering centrally driven sympathetic activity and blocking the induction of HTRS. This study provides insight into central mechanisms that exercise training inhibits the predisposition for development of hypertension when one encounters prohypertensive stressors in the lifetime. This study also highlights voluntary exercise as a non-pharmacologic therapy and management strategy for health and hypertensive individuals.

## Data Availability Statement

The raw data supporting the conclusions of this article will be made available by the authors, without undue reservation.

## Ethics Statement

The animal study was reviewed and approved by The University of Iowa and Hebei Medical University Animal Care and Use Committee.

## Author Contributions

BX, G-SZ, and AJ designed the experiments. BX, J-LC, FG, and TB performed the experiments and analyzed data. BX wrote the manuscript. BX, G-SZ, Z-GZ, and AJ revised the manuscript. All authors read and approved the final manuscript.

## Conflict of Interest

The authors declare that the research was conducted in the absence of any commercial or financial relationships that could be construed as a potential conflict of interest.

## Publisher’s Note

All claims expressed in this article are solely those of the authors and do not necessarily represent those of their affiliated organizations, or those of the publisher, the editors and the reviewers. Any product that may be evaluated in this article, or claim that may be made by its manufacturer, is not guaranteed or endorsed by the publisher.
